# A network meta-analysis of the effects of different aerobic exercise prescriptions on bone density in osteoporosis patients

**DOI:** 10.3389/fendo.2026.1828031

**Published:** 2026-06-03

**Authors:** Xiaobin Zhou, Ningning Yang, Chenlong Xi, Jing Ji, Tao Xu

**Affiliations:** 1 Department of Rehabilitation Medicine, Gansu Provincial Hospital of Traditional Chinese Medicine, Lanzhou, China; 2 College of Sports, Northwest Normal University, Lanzhou, China

**Keywords:** aerobic exercise, bone density, exercise prescription, network meta-analysis, osteoporosis

## Abstract

**Objective:**

To compare the effects of different doses of aerobic exercise on bone mineral density in osteoporosis patients through a network meta-analysis.

**Methods:**

This study systematically retrieved and screened 7 databases (including PubMed, Web of Science, and Cochrane Library, etc.) for randomized controlled trials on aerobic exercise interventions for osteoporosis patients from database inception to June 2025. The study strictly followed the PRISMA guidelines, within the framework of frequency theory, we conducted a network meta-analysis using Stata 15.0 software, quality assessment of the literature using Cochrane bias risk assessment tool.

**Results:**

A total of 27 randomized controlled trials involving 2,183 participants were included in this study. The network meta-analysis revealed that aerobic exercise regimens with the following parameter: intensity (High, medium, low-to-medium and medium-to-high intensity progressive loads), training duration (24 weeks), single-session duration (30-59min), and intervention frequency (2–3, 4–5times a week) all significantly improved bone density in osteoporosis patients compared to the control group. Indirect comparison results revealed statistically significant advantages of high-intensity exercise over moderate and low-intensity exercise. The frequency of 4–5 times a week showed a significant advantage compared to 2–3 times and 6–7 times per week. No significant differences were observed between intervention frequency and duration. The Surface Under the Cumulative Ranking Curve (SUCRA) demonstrated the following rankings: the efficacy was highest at the high intensity level, followed by the medium to high intensity level, then the low to medium intensity, the medium intensity, the Low intensity level, and the control group. For exercise duration, a 24-week intervention achieved the best efficacy, followed by a 12-week intervention, then the 48 weeks or longer, and the control group. Regarding single session intervention time, sessions lasting 30 to 59 minutes produced superior efficacy, followed by 60 minutes or longer, then the control group. In terms of weekly exercise frequency, training 4 to 5 times per week ranked first in efficacy, followed by 2 to 3 times per week, then 6 to 7 times per week, and the control group.

**Conclusion:**

The results showed that the optimal exercise dose for improving bone mineral density (BMD) in OP patients was aerobic exercise with high intensity, 30-59min per session, 4–5 times per week, and continue 24 weeks.

**Systematic Review Registration:**

https://www.crd.york.ac.uk/PROSPERO/view/CRD420251155131, identifier CRD420251155131.

## 1 Introduction

Osteoporosis (OP) is a chronic metabolic bone disease closely associated with aging, characterized by reduced bone mass and microstructural damage to bone tissue (1). With the acceleration of population aging, the resulting public health burden has become increasingly severe (2). Globally, osteoporosis affects the health of approximately 200 million people, including 10 million in the USA alone. According to statistics, in 2020, an estimated 158 million individuals aged 50 and above were at increased risk of osteoporotic fractures, and this number is projected to double by 2040 (3). In China, 46% of individuals aged 60 and above exhibit reduced bone mass, while the prevalence of osteoporosis among those over 65 reaches 32% (4). Studies indicate that approximately 80% of fractures are linked to OP (5, 6), with osteoporotic fractures occurring 3–4 times more frequently than cardiovascular diseases or cancer. Within one year after hip fracture, the disability rate reaches 50%, and the mortality rate is approximately 20% (7). More alarmingly, nearly 60% of high-risk patients with osteoporotic fractures have not received bone protection therapy (8). A model prediction suggests that implementing effective prevention and treatment interventions could reduce 24.6 million osteoporotic fractures over the next 20 years, saving billions in medical costs (2). Consequently, the prevention and treatment of osteoporosis has become a major public health issue of global concern, and it is particularly urgent to formulate reasonable and effective prevention and treatment plans and explore their scientific basis.

Exercise is currently considered the best non-pharmaceutical means to prevent and treat osteoporosis, which can comprehensively improve bone strength, muscle strength, balance, fall risk and other risk factors for fracture (9–12). The effect of exercise on osteoporosis is to increase the number of trabeculae and the thickness of bone cortex and reduce body weight. At the microscopic level, mechanical stimulation of mechanosensitive osteocytes promotes bone remodeling (13), Meanwhile, exercise stimulates skeletal muscle to secrete muscle factors, which act on muscles and bones through autocrine/paracrine mechanisms (14). Numerous exercise regimens have demonstrated efficacy in improving bone density (15), though the optimal exercise types, dosages, and personalized adaptations remain under investigation. Therapeutic exercise for osteoporosis can be categorized into two main types (16): weight-bearing aerobic exercises and resistance training using body weight or external resistance.

Compared to resistance training, aerobic exercise (AE) can ensure sufficient oxygen supply, and the exercise intensity can be accepted by most people. It is one of the simplest and easiest exercises for middle-aged and elderly people. Walking, running, dancing and other sports can produce longitudinal mechanical stimulation to the bones to promote bone formation (17). Evidence-based guidelines suggest that aerobic exercise is an effective way to increase muscle mass and strength, and is a common strategy to increase or maintain bone density in OP patients. However, the effect of AE on bone density is affected by factors such as exercise intensity, frequency and time. At present, there is still a great controversy about the efficacy of aerobic exercise dose on bone density. The Italian Osteoporosis Exercise Guidelines recommend that 30 minutes of physical activity, such as walking, is sufficient to prevent and manage osteoporosis (18). However, a systematic review indicates that exercise interventions alone are insufficient to prevent or improve bone loss in postmenopausal women (19). Razzak et al. found that high-intensity aerobic exercises (AE) with a maximum oxygen uptake exceeding 75% and heavy resistance were more effective than simple aerobic exercises (20). A randomized controlled trial on exercise for preventing and treating postmenopausal osteoporosis (PMOP) found that dynamic weight-bearing high-intensity exercises like jogging and jumping improve bone density in the hip and trochanteric region, while low-intensity aerobic exercises such as walking and tai chi enhance bone density in the spine and wrist (21). Giovanni et al. conducted a systematic rehabilitation-angle evaluation of the quality of osteoporosis guidelines published over the past decade. Exercise intensity was the most frequently mentioned characteristic in the guidelines, yet most guidelines lacked detailed specifications regarding other exercise-related features (22). Furthermore, although simple meta-analysis methods are currently available, traditional Meat analysis cannot facilitate comparisons between interventions that have never been directly compared, nor can it enable direct or indirect comparisons between different formulations of the same intervention type. To determine the most precise and specific aerobic exercise dosage for OP patients, we incorporated multiple relevant randomized controlled trials through a network meta-analysis to identify the optimal aerobic exercise intervention protocol for OP prevention and treatment.

## 2 Materials and methods

### 2.1 Protocol

This study has been registered in the International Systematic Reviews Prospective Registry (PROSPERO, registration number CRD420251155131). We conducted in accordance with the Preferred Reporting Items for Systematic Reviews and Meta-Analyses for Network Meta-Analyses (PRISMA-NMA).

### 2.2 Search strategy

We systematically searched 7 databases (PubMed, Web of Science, Embase, EBSCO, Cochrane Library, CNKI, Wanfang Date) for randomized controlled trials (RCTs) on aerobic exercise interventions for osteoporosis patients, with search periods spanning from database inception to June 2025. The search strategy combines subject headings with free terms, with key search terms including: ‘postmenopausal osteoporosis’, ‘osteoporosis’, ‘osteopenia’, ‘bone loss’, ‘bone mineral density’, ‘bone density’, ‘aerobic exercise’, ‘dancing’, ‘jogging’, and ‘walking’. Detailed search strategies for each database are provided in **Supplementary Material** (**Supplementary Table 1**).

### 2.3 Inclusion and exclusion criteria

Studies were included based on the PICOS framework as follows (1): Participants were diagnosed with osteopenia or osteoporosis, and none of the patients received hormone replacement therapy. The clinical diagnosis should meet the relevant WHO definitions, and the bone mineral density values of the study subjects should fall within this range (T-score ≤ −2.5 or −2.5 ≤ T-score ≤ −1.0) (2); Intervention: The intervention measures of the experimental group were any form and any dose of weight-bearing aerobic exercise, such as running, fast walking, skipping rope, aerobics, square dancing, etc. The specific exercise intensity, exercise frequency, exercise period and single exercise time were specified in the article (3); The control group received no exercise interventions (Low-intensity regular exercise or simple stretching exercises were also excluded) (4). Outcomes: The primary endpoint was lumbar bone mineral density, measured using dual-energy X-ray absorptiometry (DXA) or dual-photon absorptiometry (DPA) (5); Study type: RCTs.

Studies were excluded if they met any of the following criteria (1): The literature types include: reviews, conference abstracts, research proposals, and theses; studies where relevant data could not be extracted or converted from original research (even after contacting authors) (2); Studies with unclear specific exercise types or exercise plans in interventions (3); The participants who had engaged in exercise programs within the past 6 months (4); Exercise intervention cycles shorter than 8 weeks.

### 2.4 Study selection and data extraction

All retrieved references were imported into Endnote X20, where duplicates were removed. Two researchers (X.B.Zhou and S.H.Zhang) independently screened titles and abstracts based on inclusion/exclusion criteria, followed by full-text reviews of potentially eligible studies. The final selection of studies was determined through discussions between the two researchers, with a third researcher (N. Yang) involved in resolving any disputes. The extracted data included first author’s name, publication year, sample size, participant demographics (age, gender, BMI, region), prescription characteristics of aerobic exercise (intensity, frequency, duration, cycle, exercise content), and outcome indicators. All extracted information underwent double-checking, with any discrepancies resolved through group discussion. Where standard deviation (SD) was unavailable, it was calculated using standard error (SE), confidence interval (CI), or t-values and p-values. Alternatively, authors were contacted via email at least three times to supplement missing data.

### 2.5 Assessment of risk of bias

This study employed the Cochrane Risk of bias 1.0 (RoB1) assessment Tool. The assessment criteria included seven items: randomization sequence generation, concealed assignment, patient and investigator blinding, evaluator blinding, incomplete outcomes (i.e., withdrawals), selective reporting of results, and other bias risks. Each item was rated as “low risk,” “high risk,” or “uncertain.” Disagreements were resolved through discussion or judgment by a third researcher. The literature bias assessment was conducted using the Rev Man 5.4 software.

### 2.6 Transitivity hypothesis, consistency testing, and model selection

Transitivity is the core prerequisite of network meta-analysis. This study ensured transitivity through the following measures: comparability of study populations: all subjects met the WHO diagnostic criteria for osteoporosis/osteopenia (T-value ≤ -2.5 or-1.0 ≤ T-value ≤ -1.0), did not receive hormone replacement therapy, and exhibited balanced baseline age, gender, BMI, and bone mineral density levels across studies. Intervention comparability: The experimental group underwent weight-bearing aerobic exercise, with clearly reported intensity, frequency, duration per session, and total duration; the control group received no exercise intervention, with identical protocol definitions. Comparable outcome measures: The primary outcome was lumbar bone mineral density (BMD), with standardized measurement methods and consistent units. Comparability of study design: All were RCTs, with similar follow-up durations and outcome measurement protocols. Consistency is assessed through a combination of global inconsistency testing, node splitting method, and circular inconsistency testing. When P> 0.05, no significant inconsistency is detected, and the consistency model is employed for analysis.

### 2.7 Statistical analysis

In accordance with the PRISMA network meta-analysis guidelines, a random-effects model was applied within the frequency framework, with effect size aggregation and 95%CI calculated using Stata 15.0 software. Given the uniform measurement units across outcome indicators, mean difference (MD) was used as the aggregated effect size (23–25). The network evidence diagram illustrates the relationships among exercise interventions, where connecting lines represent direct comparisons between interventions. The line thickness corresponds to the number of studies, while node size reflects the total sample size of the corresponding intervention. The area under the cumulative rank probability diagram (SUCRA) was used to rank and compare the intervention effects of different exercise types, where a larger area indicates a higher ranking. Funnel plots were used to test for publication bias or small sample effect.

The classification of exercise intensity levels is based on prior research (26, 27), and the classification of exercise intensity was established by converting the intensity grading criteria for physical activity from the World Health Organization (WHO) 2020 Physical Activity Guidelines into maximum heart rate values (28). categorizing AE into high-intensity (>75% HRmax or greater than 6 METs), moderate-intensity (60%-75% HRmax or 3-6METs), low-intensity (<60% HRmax or 1.5–3 METs), and progressive load intensity (Low to moderate intensity, and moderate to high intensity). The intervention effects of these AE intensity levels are ranked. Other outcome indicators are classified according to their respective ranges.

## 3 Results

### 3.1 Selection process and characteristics of included studies

**Figure 1:**
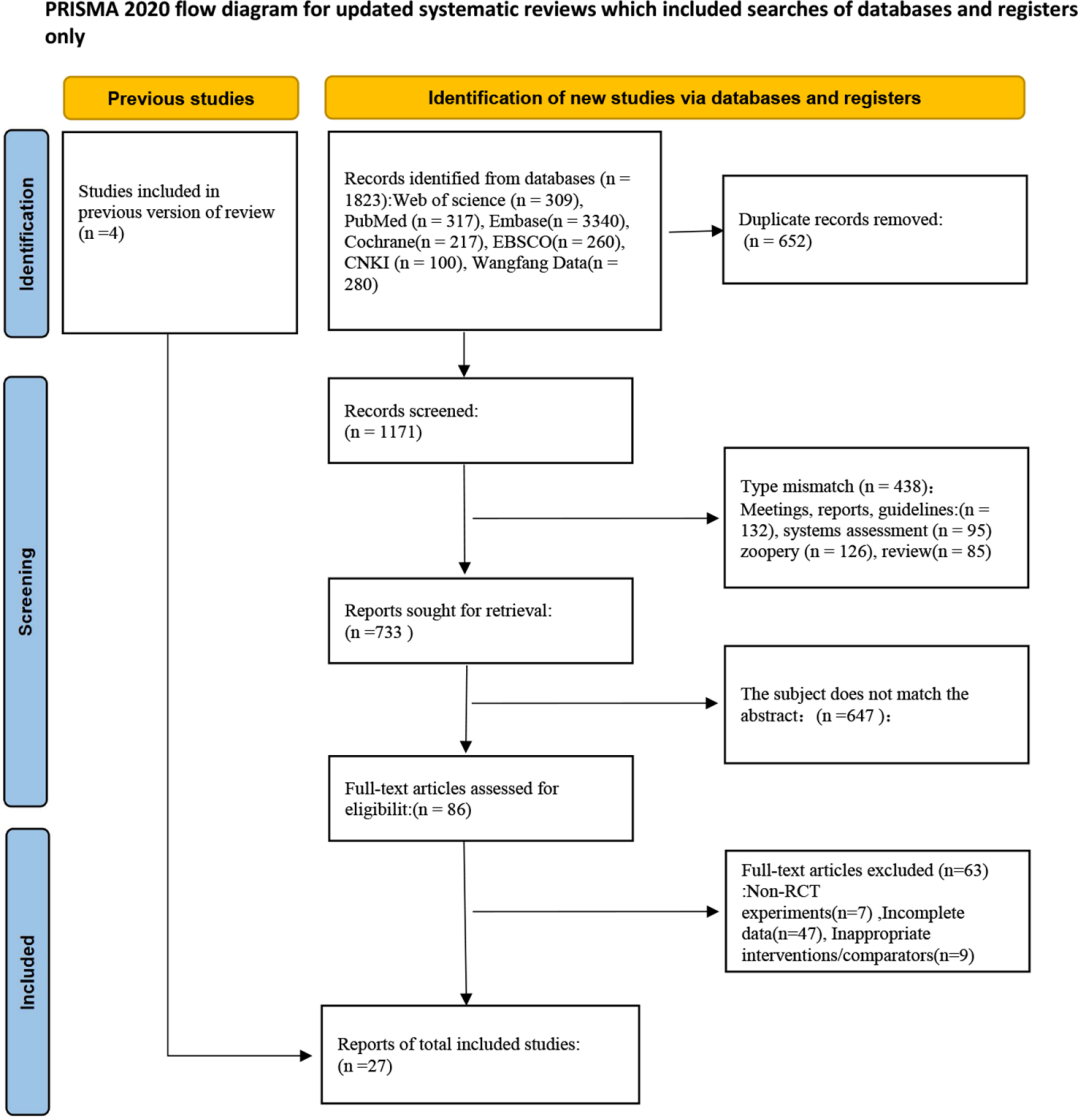
PRISMA flow diagram of the study selection process.

A total of 1,823 relevant articles were retrieved, and 86 studies underwent full-text evaluation through a multi-level screening process. Following comprehensive assessment based on inclusion/exclusion criteria, 27 RCTs meeting the inclusion criteria were ultimately included in the network meta-analysis (**Figure 1**). Involving a total sample size of 2,183 participants (1,120 in the intervention group and 10,63 in the control group). The prescription characteristics of aerobic exercise included exercise intensity (measured as maximum heart rate), duration of single sessions, weekly intervention frequency, and intervention cycles (**Table 1**).

**Table 1:** Study characteristics.

Study	Sample		Age(y)	Characteristics of exercise intervention				
	T	C		Intensity (HRmax)	Weeks	Time (min)	Frequency (times/W)	Content
Nie 2019 (39)	10	10	64.6 ± 4.4	60%-80%	12	60	3	Brisk walking
iYamazaki 2004 (29)	15	27	64.2 ± 2.9	60%-70%	48	60	4	Brisk walking
Zhou 2020 (57)	82	82	51.50 ± 2.8	70%	48	60-100	3	Jogging
Peng 2016 (35)	20	20	53.9 ± 4.9	80%-90%	24	30-35	4	Brisk walking
	20		53.2 ± 5.1	60%-80%	24	30-35	4	Brisk walking
	20		52.2 ± 5.7	35%-60%	24	30-35	4	Brisk walking
Sun R.X 2012 (30)	30	30	54.7 ± 5.8	70%-80%	72	>30	5	Jogging, aerobics
LI N.J 2019 (58)	96	96	64.1 ± 8.27	60%-70%	48	30	7	walking, aerobics
Qin Jinze 2017 (59)	25	25	45~60	60%-70%	24	30-60	5	Square dance
Wang Y.C.2018 (60)	84	84	68.3 ± 6.6	60%-70%	48	30-60	5	Square dance
Qin 2018 (61)	25	25	45~60	60%-70%	24	30-60	5	Square dance
Pei-AnYu 2019 (62)	40	40	62.5 ± 6.6	50%-70%	24	60	3	Aerobic dancing
Gao sheng 2017 (63)	30	30	50~60	70%-80%	48	45	3-5	Jogging
Hu Y 2017 (64)	20	20	70.2 ± 3.69	55%-75%	24	30-60	5	Jogging
Liu M 2018 (65)	40	40	40.7 ± 6.2	70%-80%	24	30	3	Jogging
Song J.L2018 (66)	32	31	65.3 ± 3.0	55%-65%	48	50	5	Square dance
Huang Z.H2016 (67)	20	20	55.98 ± 3.43	60%-70%	24	90	3	Square dance
Chen.L.H2024 (36)	48	47	70.71 ± 4.72	50%-70%	24	30-60	3-5	Walking, jogging
Sun J.C2009 (68)	30	30	63 ± 1.42	70%-85%	48	30-60	4-6	Aerobics
Tartibian 2011 (37)	20	18	59.7 ± 2.3	45%-65%	24	35	3-6	Walking, jogging
M. Y.Chien2000 (31)	22	21	57.1 ± 8.6	80%-90%	24	40	3	Stepping, walking
Gina Bravo1996 (38)	61	63	59.6 ± 5.82	70%-85%	48	60	3	Walking, dancing
Gina Bravo1997 (32)	77	77	59.4 ± 5.51	65%-75%	48	60	3	Skipping
Iwamoto1998 (69)	32	36	64.8 ± 6.1	75%-85%	24	60	2	Walking
Yang Y.F2020 (70)	45	45	73.0 ± 1.43	55%-65%	12	60	5	Walking
Zhu H.L2007 (71)	48	48	67.1 ± 7.2	70%-80%	48	30-60	3	Walking, jogging
Ruan C.L2016 (33)	30	30	55.4 ± 5.7	60%-80%	12	30	3	Jogging, dancing
	30		55.0 ± 5.9		24	30-60	3	
Hou H.b2015 (72)	43	43	68.0 ± 3.9	70%-85%	48	30-60	4-6	Climb mountains
Alayat2017 (73)	25	25	54.2 ± 3.09	40%-60%	48	60	3	Climb stairs and jog

T, treatment group, C, control group.

### 3.2 Risk of bias assessment

**Figure 2:**
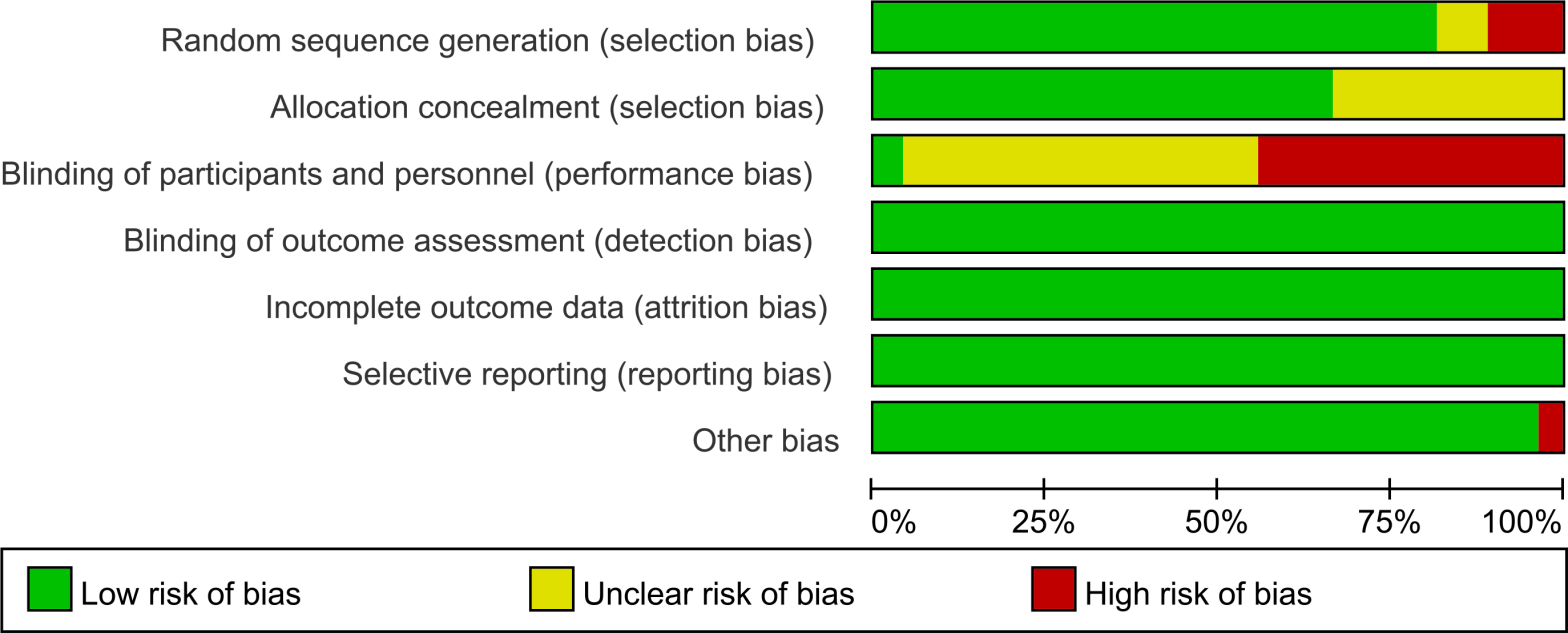
Risk of bias graph.

The bias risk assessment results indicate (**Figure 2**; detailed bias risk evaluations for each study are provided in **Supplementary Figure 1**). Three studies assigned participants to groups based on their own preferences (29–31), one study did not specify the grouping method (32), and the remaining studies using randomization were rated as low risk. In the distribution of hidden items, two methods are not specified (32, 33). Due to the nature of the intervention, clinical studies require ethical review and informed consent from participants. However, implementing blinding for both subjects and researchers is often challenging, resulting in only one study being double-blind (34). Although many studies did not specify the blinding of the result evaluators, most of them were operated by professional technicians to measure the instrument, and the reliability of the results was good, so the blinding of the result measurement was rated as low risk. Overall, most studies demonstrated adequate data completeness, eight studies reported participant loss, with seven studies showing similar inter-group loss patterns and causes that had no significant impact on results (29, 31, 35–38). Selective reporting was assessed as low risk. One study had a sample size of 10 per group, potentially introducing small sample bias (39).

### 3.3 Network meta-analysis

#### 3.3.1 Network evidence plot and consistency analysis

**Figure 3:**
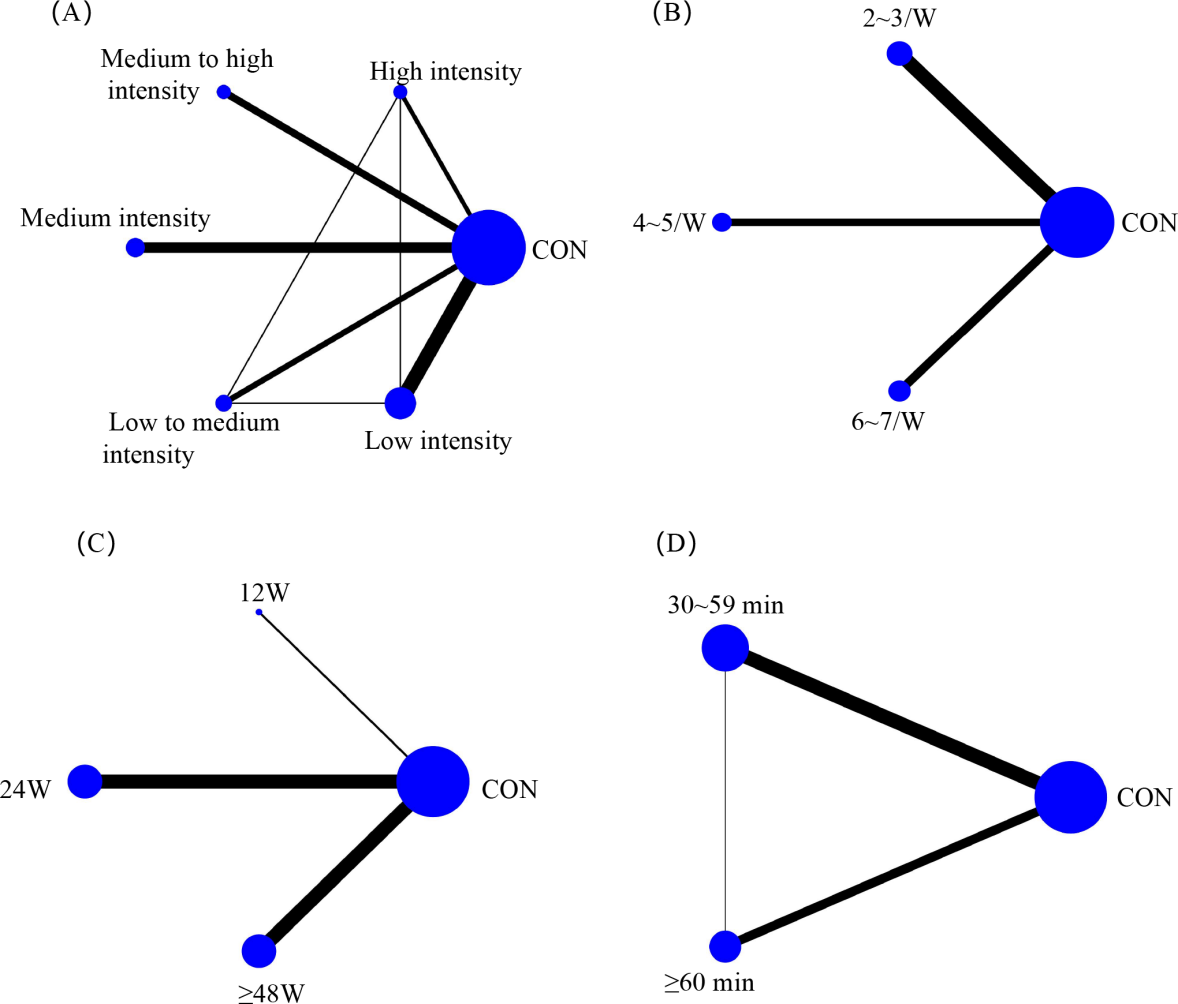
Network evidence plot. **(A)** Intensity, **(B)** Frequency, **(C)** Period of motion, **(D)** Time.

The network relationships among the studies (**Figure 3**) revealed closed loops in two outcome indicators: exercise intensity and duration of single exercise sessions. Global inconsistency test, node-splitting test and loop inconsistency test were performed for outcomes with closed loops. Results showed no significant inconsistency for all outcomes (all *P > 0.05*), suggesting high agreement between direct and indirect evidence and satisfying the consistency assumption. Therefore, a consistency model was used for all network meta-analyses, ensuring reliable results.

#### 3.3.2 Direct comparison versus indirect comparison

**Figure 4:**
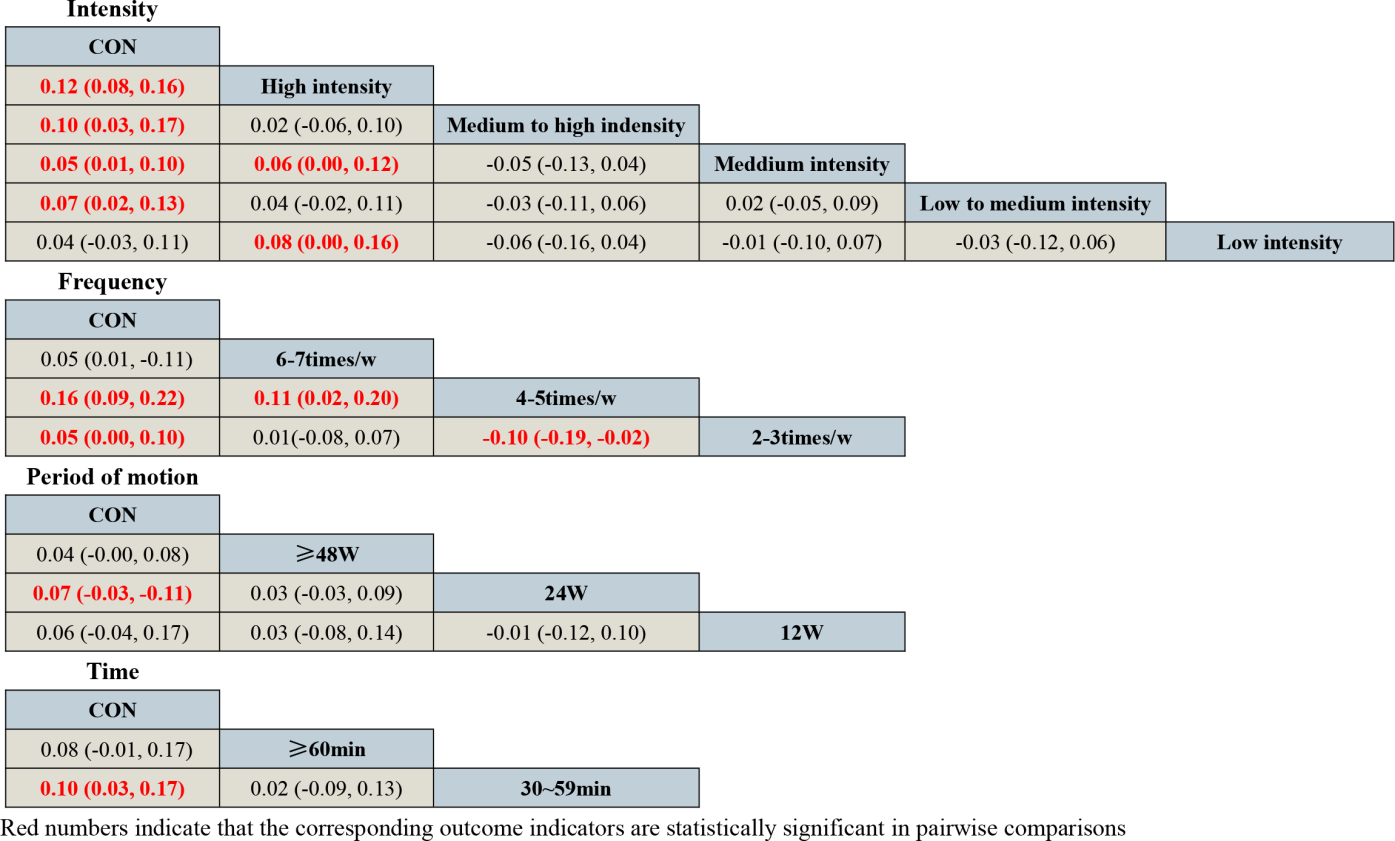
League plot of the network meta-analysis of various outcome indicators.

The direct comparison results show that (**Figure 4**). Compared with the control group, high intensity AE [MD = 0.12, 95%CI (0.08, 0.16)], medium-to-high intensity AE [MD = 0.10, 95%CI (0.03, 0.17)], medium intensity AE [MD = 0.05, 95%CI (0.01, 0.10)], and low-to-medium intensity AE [MD = 0.07, 95%CI (0.02, 0.13)] showed significant improvements. Additionally, AE with intervention frequencies of 2–3 times/week [MD = 0.05, 95%CI (0.00, 0.10)] and 4–5 times/week [MD = 0.16, 95%CI (0.09, 0.22)], a 24 weeks training cycle [MD = 0.07, 95%CI (0.03, 0.11)], and single-training sessions lasting 30–59 min [MD = 0.10, 95%CI (0.03, 0.17)] were all significantly effective in improving BMD in OP patients.

The indirect comparison results showed that high intensity AE had a significant advantage over low intensity AE [MD = 0.08, 95%CI (0.00,0.16)] and medium intensity AE [MD = 0.06, 95%CI (0.00,0.12)]. Compared with 6–7 times/week [MD = 0.11, 95%CI (0.02,0.20)] and 2–3 times/week [MD = 0.10, 95%CI (0.02,0.19)] AE, 4–5 times/week exercise frequency showed a significant advantage, while other prescription characteristics showed no significant differences (**Figure 4**).

#### 3.3.3 Best intervention prescription probability ranking results

**Figure 5:**
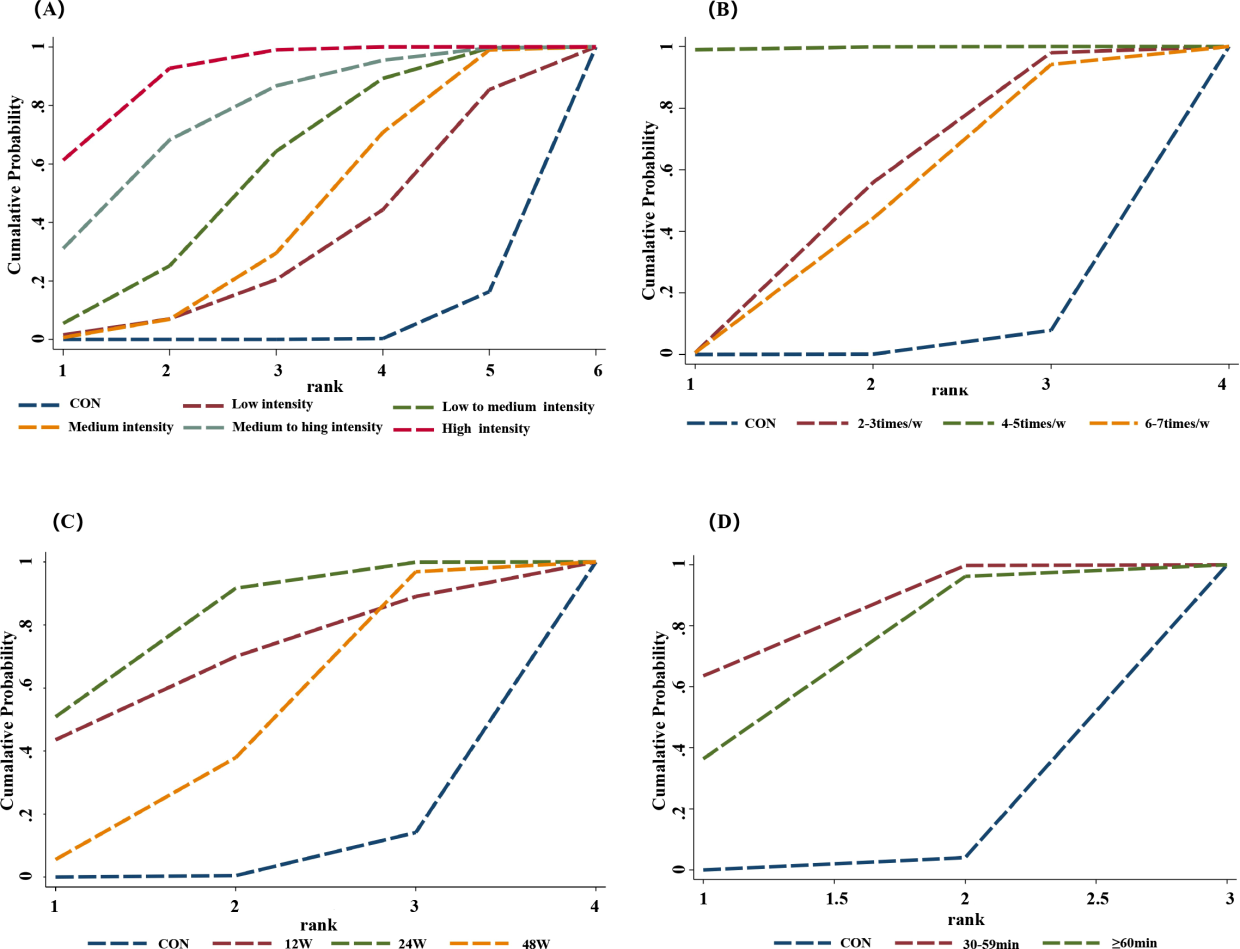
Probability ranking plot of intervention effects. **(A)** Intensity, **(B)** Frequency, **(C)** Period of motion, **(D)** Time.

The probability-based ranking results (**Figure 5**) indicate that aerobic exercise intensity is ranked in descending order as follows: high intensity (SUCRA = 90.6%) >medium to high intensity (SUCRA = 76.3%) > low to medium intensity (SUCRA = 56.8%) >medium intensity (SUCRA = 42.8%%) > low-intensity (SUCRA = 31.6%) >CON (SUCRA = 3.4%). The effectiveness ranking of weekly exercise frequency was as follows: 4–5 times/w (SUCRA = 99.6%) > 2–3 times/w (SUCRA = 51.5%) > 6–7 times/w (SUCRA = 46.3%) > CON (SUCRA = 2.6%). The efficacy of exercise regimens ranked as follows: 24 weeks (SUCRA = 80.8%) > 12 weeks (SUCRA = 67.5%) > 48 weeks or longer (SUCRA = 46.8%) > CON (SUCRA = 4.9%). The effect of single exercise duration was ranked as follows: 30–59 minutes (SUCRA = 81.7%) > 60 minutes and above (SUCRA = 66.3%) > CON (SUCRA = 2.0%).

### 3.4 Publication bias analysis

**Figure 6:**
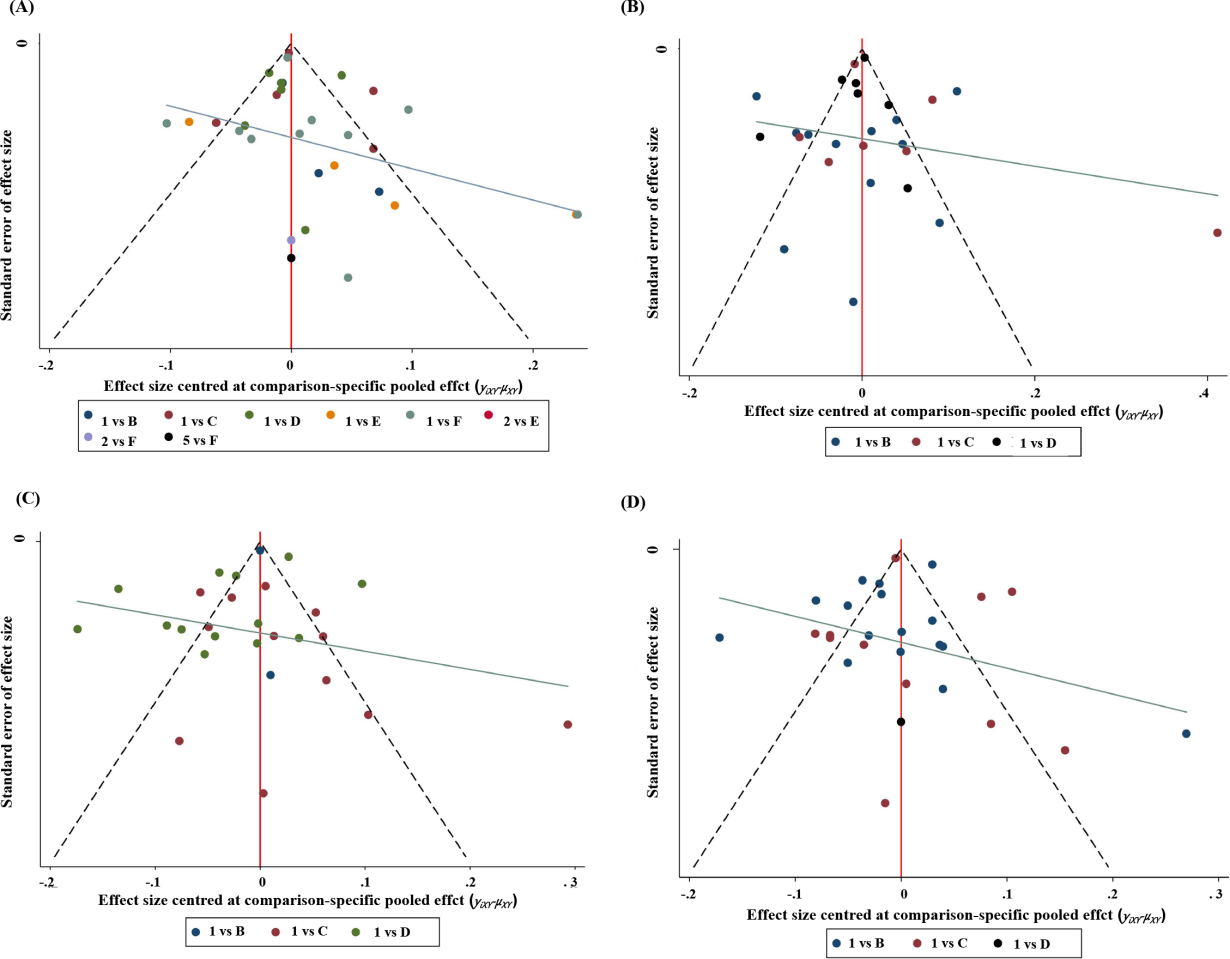
Funnel plot. **(A)** Intensity, **(B)** Frequency, **(C)** Period of motion, **(D)** Time.

Publication bias was examined using funnel plots for outcome measures, which showed good symmetry in the funnel plots of each outcome measure (**Figure 6**), with little impact from publication bias or small sample effects.

## 4 Discussion

This study conducted a network meta-analysis of 27 RCTs meeting inclusion criteria to compare the effects of different aerobic exercise intensities on bone density, ultimately identifying the optimal aerobic exercise regimen for improving lumbar bone density in patients with primary osteoporosis.

Clinical guidelines for osteoporosis prevention and management recommend exercise training as an effective method to maintain bone mass or slow bone loss throughout menopause and old age. However, not all forms or doses of exercise training are equally effective in triggering positive skeletal responses. Exercise intensity, as a key parameter in aerobic exercise prescriptions, directly impacts the degree of bone density improvement and intervention efficacy in osteoporosis patients. The results of this study show that low-intensity aerobic exercise cannot effectively improve bone density in osteoporosis patients, while the other four intensity levels of aerobic exercise can significantly improve bone density, with high-intensity aerobic exercise demonstrating the most significant effects. This aligns with previous research findings (19), previous studies indicate that brisk walking at 75% or higher of maximum oxygen uptake (40, 41), when combined with weighted vests or other activities like jogging, stair climbing, or step-counting, can provide partial protection against bone loss. This is because high-intensity exercise produces greater mechanical load, which can more effectively stimulate osteoblast activity and promote bone formation. Meanwhile, the hormonal response triggered by high-intensity exercise (such as increased secretion of growth hormone and IGF-1) is more pronounced (42, 43). A logistic regression analysis of osteoporosis risk factors involving approximately 7,000 participants revealed that high-impact exercise is associated with reduced osteoporosis risk. Compared to sedentary individuals, high-intensity exercise significantly decreases the likelihood of developing osteoporosis. Conversely, low-intensity exercise showed no significant association with osteoporosis, particularly among men (44). A RCT (Peng, 2016) compared three different intensities of aerobic exercise. Compared with the control group, the low-intensity aerobic exercise group showed no significant change in lumbar bone mineral density, nor any marked improvement compared to baseline levels (35).

Although our study has confirmed that high-intensity aerobic exercise significantly improves bone mineral density in patients with osteoporosis, great attention must be paid to exercise safety and the appropriate target population. High-intensity exercise is associated with increased heart rate, elevated cardiopulmonary load, and greater mechanical stress on the lower limbs. For high-risk patients it may increase the risks of falls, cardiovascular stress, and fractures. progressive intensity strategies, with moderate-intensity sustained activities like brisk walking and Tai Chi being more appropriate (45). Although low-intensity exercise provides minimal mechanical stimulation to bones, it positively impacts cardiovascular function, muscle endurance, and motor agility, indirectly enhancing bone health. Long-term outdoor walking demonstrates a significant negative correlation with osteoporosis risk, particularly evident in individuals with low genetic susceptibility. Current research indicates that regular light-to-moderate walking significantly improves pain, walking speed, balance, lower limb stability, and daily functioning in postmenopausal women with osteopenia compared to inactive individuals (21).The importance of outdoor walking as a simple and economical adjunct to public health programs for osteoporosis prevention (46). Prior to clinical application, stratified assessments of fall risk, cardiopulmonary function, and fracture risk should be conducted. High-risk or debilitated patients should avoid initiating high-intensity interventions; instead, a progressive intensity escalation strategy under professional supervision is recommended, starting with low-to-moderate intensity and gradually adjusting frequency and duration.

The effect of intervention period on bone mineral density follows the principle of diminishing returns (19, 47), After any exercise-induced initial skeletal adaptation, this response will eventually fade over time, and subsequent gains will usually be slow or stop. However, our study demonstrated that a 24-week exercise intervention produced better outcomes than a 12-week one. An important factor explaining this phenomenon is that although the benefits of skeletal response to mechanical stimulation diminish over time, the response of bones to load is slower because the typical bone remodeling cycle lasts 12 to 32 weeks (48), interventions take 24 to 36 weeks to pay off. Results of multiple exercise interventions over 12 to 18 months showed the greatest BMD change occurred in the first 5 to 6 months (49). Some studies have reported a linear increase in bone mineral density with sustained exercise training (50). This may be attributed to the persistent overload caused by progressive exercise regimens and the bones’ continuous adaptation to new stress stimuli. Our findings indicate that 48-week or longer exercise interventions showed lower lumbar bone mineral density improvements in subjects compared to 12-week and 24-week interventions in the SUCRA rankings. This discrepancy stems from the original 48-week study, where most exercise intensities were moderate with limited progression, resulting in minimal skeletal changes. Overall, this study demonstrates an inverted U-shaped relationship between intervention duration and lumbar bone mineral density in OP patients. This phenomenon aligns with the classical pharmacological and exercise physiology theory of dose-response relationships (51, 52), which posits that external interventions do not provide indefinite benefits but rather an optimal dose range where efficacy peaks. Benefits diminish when interventions fall below or above this range. To achieve optimal outcomes in long-term sustained interventions, it is essential to vary stimuli (e.g., intensity progression or exercise content transitions) rather than maintaining a fixed intensity level.

Furthermore, decreased patient adherence may occur during ultra-long-term exercise interventions (e.g., ≥48 weeks), which is a key factor contributing to the decline in long-term efficacy, due to both physiological and psychological responses in elderly individuals. The adherence challenges associated with prolonged intervention durations also stem from post-exercise joint discomfort and cumulative exercise fatigue, leading to reduced willingness and ability to maintain consistent exercise. When developing exercise prescriptions for postmenopausal osteoporosis, highly supervised aerobic exercise regimens should be adopted, with adherence maintained through increased engagement to prevent efficacy deterioration caused by excessively long intervention durations.

Bone gains from exercise require consistent time and frequency to maintain. The minimum effective dose for long-term positive bone effects is at least two weekly sessions (53), once this cumulative bone effect is stopped it will disappear. In the studies included in this article, only one intervention group received 2 sessions per week, while others had ≥3 sessions. Among those who maintained aerobic exercise at least 4 times weekly, the improvement in lumbar and femoral neck bone density was significantly greater than in low-frequency exercise groups. A meta-analysis on osteoporosis exercise frequency indicated that higher net training frequency yields better results for BMD. Osteoporosis exercise programs should provide at least 3 sessions per week, allowing net training frequency to exceed two sessions per week except for absences (54),and aerobic exercise for 30–60 minutes, 4–7 times a week for 6 months can significantly increase bone density in patients with osteoporosis. HEJAZI et al. also demonstrated that aerobic exercise performed more than three times weekly yields better bone density improvement in middle-aged and elderly populations, particularly in the lumbar spine and greater trochanter regions (55). From a physiological point of view, this frequency provides sufficient mechanical stimulation to continuously activate the bone remodeling process, while allowing adequate recovery time to avoid increased bone resorption due to overtraining.

The Rehabilitation Working Group of the International Osteoporosis Foundation (IOF) has demonstrated that regular physical activity (PA), including moderate-to-high-intensity weight-bearing PA and resistance training, is consistently associated with higher bone mineral density and reduced fracture risk in adults and older adults. Even mild PA replaces sedentary behavior and provides measurable benefits for bone health, particularly in older adults and postmenopausal women, potentially contributing to a lower fracture risk (56), this is highly consistent with our results. In conclusion, we advocate that promoting physical activity (PA) while reducing osteoporosis should be a core focus of clinical practice and public health policies, aimed at maximizing and maintaining bone health, preventing osteoporotic fractures, throughout the entire life cycle.

While we have maintained rigorous scientific standards in our research design and statistical methods, this study has certain limitations and shortcomings (1): Due to the intervention nature, the included studies could not implement blinded methods for participants and researchers, which may introduce potential bias (2). This analysis exclusively focused on studies quantifying exercise intensity through maximum heart rate measurement, excluding indicators such as maximal oxygen uptake (3). Limited by the original research volume, we did not categorize participants by gender, age and different types of aerobic exercises(e.g., dancing, running, and walking). This was because most studies did not explicitly report gender ratios, with some studies including both male and female participants. Regarding age, the included studies spanned a wide age range. Categorizing participants by gender and age might have resulted in insufficient sample sizes for each subgroup, thereby compromising the robustness of the evidence.

## 5 Conclusion

In general, the exercise prescription with high intensity(it is best to increase the intensity gradually), medium frequency (4–5 times per week), 30–59 minutes per session, and 24 weeks of continuous exercise may be the most effective aerobic exercise prescription to improve bone mineral density in patients with osteoporosis or reduced bone mass. However, to ensure the safety and tolerability of the subjects, it is recommended to adopt a progressive increase in exercise intensity.

## Data Availability

The datasets presented in this study can be found in online repositories. The names of the repository/repositories and accession number(s) can be found in the article/**Supplementary Material**.
