# Supplementary material for: A network meta-analysis of the effects of different aerobic exercise prescriptions on bone density in osteoporosis patients
Source: Front Endocrinol (Lausanne). 2026 Jun 3;17:1828031. doi: 10.3389/fendo.2026.1828031 (PMC13271876; doi:10.3389/fendo.2026.1828031)

**Supplementary Material**

**Table S1.** Detailed searching strategies

(1) Search strategy in PubMed（n = 317）

| #1 | (Osteoporosis OR osteopenia[MeSH Major Topic]) OR (Bone Loss[Title/Abstract] OR Bone mass[Title/Abstract] OR Postmenopausa Osteoporosis[Title/Abstract] OR Osteoporoses[Title/Abstract]) |
| --- | --- |
| #2 | (Aerobic exercise[MeSH Major Topic]) OR (Dancing[Title/Abstract] OR Walking[Title/Abstract] OR mountain climbing[Title/Abstract] OR Fast walking[Title/Abstract] OR jogging[Title/Abstract] OR Aerobic exercise[Title/Abstract] OR skipping[Title/Abstract]) |
| #3 | (Bone Mineral Density[MeSH Major Topic]) OR (Bone mineral Content OR Bone Mineral Density OR bone density[MeSH Major Topic]) |
| #4 | Randomized controlled trial |
| #5 | #1 AND #2 AND #3 |

(2) Search strategy in Embase（n = 340）

| #1 | osteoporosis:ti,ab,kw OR osteopenia:ti,ab,kw OR osteoporoses:ti,ab,kw OR 'age-related osteoporoses':ti,ab,kw OR 'bone loss':ti,ab,kw OR 'post-traumatic osteoporoses':ti,ab,kw OR 'postmenopausa osteoporosis':ti,ab,kw |
| --- | --- |
| #2 | 'bone densities':ti,ab,kw OR 'density, bone':ti,ab,kw OR 'bone mineral density':ti,ab,kw OR 'bone mineral densities':ti,ab,kw OR 'bone mineral contents':ti,ab,kw OR 'bmd':ti,ab,kw |
| #3 | 'aerobic exercises':ti,ab,kw OR 'physical activity':ti,ab,kw OR dancing:ti,ab,kw OR walking:ti,ab,kw OR 'mountain climbing':ti,ab,kw OR 'fast walking':ti,ab,kw OR jogging:ti,ab,kw OR 'aerobic exercise':ti,ab,kw OR 'rope skipping':ti,ab,kw OR hitt:ti,ab,kw |
| #4 | randomized AND controlled AND trial |
| #5 | #1 AND #2 AND #3 |

(3) Search strategy in Web of science（n = 309）

| #1 | TS=(“Osteoporosis” OR “Osteoporoses” OR Age-Related Osteoporoses OR Bone Loss OR Post-Traumatic Osteoporoses OR Postmenopausa Osteoporosis) |
| --- | --- |
| #2 | TS=(“Aerobic Exercises” OR Physical Activity OR Dancing OR Walking OR mountain climbing OR Fast walking OR jogging OR Aerobic exercise OR skipping OR HITT) |
| #3 | TS=(Bone Densities OR Density, Bone) OR (“Bone Mineral Density”) OR (Bone Mineral Densities OR Bone Mineral Contents) OR (“BMD”) |
| #4 | TS=Randomized controlled trial |
| #5 | #1 AND #2 AND #3 AND #4 |

(4) Search strategy in Cochrane Library（n = 217）

| #1 | MeSH descriptor: [Osteoporosis] explode all trees |
| --- | --- |
| #2 | ("osteoporoses"):ti,ab,kw OR (Age-Related Osteoporoses):ti,ab,kw OR (Bone Loss):ti,ab,kw OR (Postmenopausa Osteoporosis):ti,ab,kw OR ("osteopenias"):ti,ab,kw |
| #3 | (osteoporosis):ti,ab,kw |
| #4 | #1 OR #2 OR #3 |
| #5 | MeSH descriptor: [Exercise] explode all trees |
| #6 | ("aerobic exercise"):ti,ab,kw OR ("physical activity"):ti,ab,kw OR ("dancing"):ti,ab,kw OR ("walking distance"):ti,ab,kw OR ("jogging"):ti,ab,kw |
| #7 | (mountain climbing):ti,ab,kw OR (rope skipping):ti,ab,kw OR (HITT):ti,ab,kw |
| #8 | #5 AND #6 OR #7 |
| #9 | MeSH descriptor: [Bone Density] explode all trees |
| #10 | (Density, Bone):ti,ab,kw OR (“Bone Mineral Density”):ti,ab,kw OR (bone-mineral densities):ti,ab,kw OR (Bone Mineral Contents):ti,ab,kw OR (BMD):ti,ab,kw |
| #11 | (bone density):ti,ab,kw |
| #12 | #9 OR #10 OR #11 |
| #13 | #4 OR #8 OR #12 |

(5) Search strategy in EBSCO（n = 260）

| #1 | SU = (Osteoporosis OR osteopenia OR Osteoporoses OR Age-Related Osteoporoses OR Bone Loss OR Post-Traumatic Osteoporoses OR Postmenopausa Osteoporosis) |
| --- | --- |
| #2 | SU = (Aerobic Exercises OR Dancing OR Walking OR mountain climbing OR Fast walking OR jogging OR Aerobic exercise OR rope skipping OR HITT) |
| #3 | SU = (Bone Densities OR Density, Bone OR “Bone Mineral Density” OR Bone Mineral Densities OR Bone Mineral Contents OR “BMD”) |
| #4 | #1 OR #2 OR #3 |

(6) Search strategy in CNKI（n = 100）

| #1 | 主题=（骨质疏松 + 骨质疏松症+骨量减少） AND 主题=（有氧运动 + 有氧运动治疗 + 有氧运动训练） AND 主题=（骨密度） |
| --- | --- |

(7) Search strategy in Wanfang Data（n = 280）

| #1 | 题名或关键词=（骨质疏松 OR 骨量减少 OR 绝经后骨质疏松） AND 主题=（有氧运动 OR 步行 OR 跑步 OR 爬山 OR 舞蹈 OR 健身操） AND 提名或关键词=（骨密度） |
| --- | --- |

**Figure S1.** Result of the risk of bias assessment


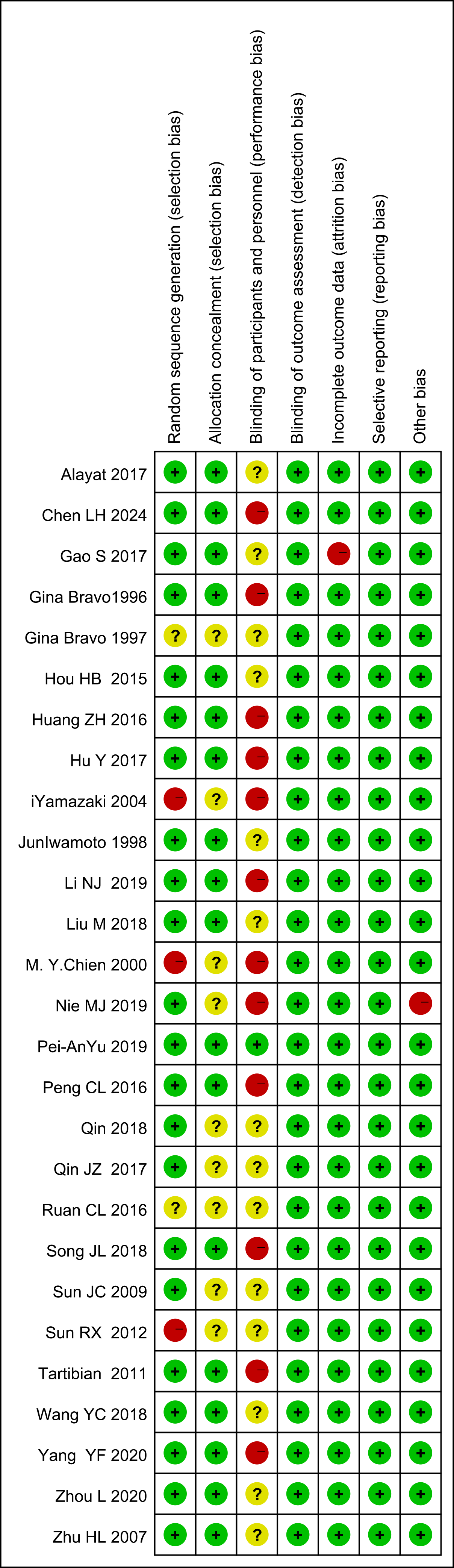

Supplement: Supplementary file 1 [file DataSheet1.docx]
